# The role of the C-terminal tail region as a plug to regulate XKR8 lipid scramblase

**DOI:** 10.1016/j.jbc.2024.105755

**Published:** 2024-02-15

**Authors:** Takaharu Sakuragi, Ryuta Kanai, Mayumi Otani, Masahide Kikkawa, Chikashi Toyoshima, Shigekazu Nagata

**Affiliations:** 1 Laboratory of Biochemistry and Immunology, World Premier International Immunology Frontier Research Center, Osaka University, Suita, Osaka, Japan; 2 Institute for Quantitative Biosciences, The University of Tokyo, Tokyo, Japan; 3 Department of Cell Biology and Anatomy, Graduate School of Medicine, The University of Tokyo, Tokyo, Japan

**Keywords:** apoptosis, cryo-EM, membrane protein, phospholipids, protein structure, scramblase

## Abstract

XK-related 8 (XKR8), in complex with the transmembrane glycoprotein basigin, functions as a phospholipid scramblase activated by the caspase-mediated cleavage or phosphorylation of its C-terminal tail. It carries a putative phospholipid translocation path of multiple hydrophobic and charged residues in the transmembrane region. It also has a crucial tryptophan at the exoplasmic end of the path that regulates its scrambling activity. We herein investigated the tertiary structure of the human XKR8–basigin complex embedded in lipid nanodiscs at an overall resolution of 3.66 Å. We found that the C-terminal tail engaged in intricate polar and van der Waals interactions with a groove at the cytoplasmic surface of XKR8. These interactions maintained the inactive state of XKR8. Point mutations to disrupt these interactions strongly enhanced the scrambling activity of XKR8, suggesting that the activation of XKR8 is mediated by releasing the C-terminal tail from the cytoplasmic groove. We speculate that the cytoplasmic tail region of XKR8 functions as a plug to prevent the scrambling of phospholipids.

Phospholipids are asymmetrically distributed in the lipid bilayer of the plasma membrane (1). Phosphatidylserine (PtdSer) and phosphatidylethanolamine localize exclusively to the inner leaflet of the plasma membrane, whereas phosphatidylcholine and sphingomyelin (SM) are mostly present in the outer leaflet. This asymmetrical distribution of phospholipids is maintained by flippases (P4-ATPases), which translocate anionic phospholipids (PtdSer and phosphatidylethanolamine) from the external leaflet to the inner leaflet at the cost of ATP (2, 3).

The asymmetrical distribution of phospholipids is disrupted in various cells, such as apoptotic cells, activated platelets, and activated lymphocytes, which expose PtdSer on their surface to activate enzymes or as a signaling molecule (3, 4, 5). PtdSer exposed on apoptotic cells functions as an “eat me” signal for phagocytes and is essential for the efficient clearance of apoptotic cells (6, 7). Flippases at plasma membranes are inactivated when cells expose PtdSer on their surface (2, 8). However, since it is energetically unfavorable for phospholipids to spontaneously move to the other leaflet across the hydrophobic core of the membrane (9), the inactivation of flippases is not sufficient for cells to quickly expose PtdSer. Scramblases that nonspecifically and bidirectionally translocate phospholipids between lipid bilayers are required for the efficient exposure of PtdSer on the cell surface (3).

We previously identified two scramblases, the calcium-activated scramblase TMEM16F and the caspase-dependent scramblase XK-related 8 (XKR8) (10, 11). XKR8 belongs to the XKR family, in which XKR4, XKR8, and XKR9 are activated by caspase to function as scramblases (12). XKR8 forms a heterodimer with basigin (BSG) or neuroplastin and requires either for its localization to the plasma membrane (13). XKR8 is activated during apoptosis when it is cleaved by caspase at the conserved recognition site in its C-terminal cytoplasmic tail (11). Since apoptotic cells lacking *Xkr8* do not expose PtdSer and are not efficiently engulfed by macrophages, *Xkr8*-deficient mice develop systemic lupus erythematosus–type autoimmune disease or male infertility (14, 15). XKR8 is also activated in living cells by the kinase-mediated phosphorylation of residues in the C-terminal tail region downstream of the caspase cleavage site (16).

We and others elucidated the resting state structures of the human XKR8 (hXKR8)–BSG complex and rat XKR9 in detergent micelles, which have similar arrangements with eight transmembrane helices and two helices that penetrate halfway to the membrane (17, 18). The structure of hXKR8 and its mutational analysis revealed a hydrophobic cleft on the lipid-exposed surface of the molecule to recruit phospholipids, an intramolecular phospholipid path of several hydrophilic residues, and a crucial tryptophan (Trp45) at the extracellular end of the track that regulates its scrambling activity (17).

Integral membrane proteins, including scramblases, interact in the lipid bilayer with lipids that change the structural or regulatory properties of membrane proteins (19, 20, 21, 22). To visualize the structures of XKR8 in a lipid environment, we herein performed a cryo-EM single-particle analysis of the hXKR8–BSG complex embedded in lipid nanodiscs. We obtained a density map of the hXKR8–BSG complex in lipid nanodiscs and presented a revised atomic model of the C terminus including the amphipathic helix (α11). The hXKR8–BSG complex has a groove at the cytoplasmic surface. The C-terminal cytoplasmic tail bound to the groove through polar and van der Waals interactions, and the disruption of these interactions strongly enhanced scrambling activity.

## Results

### Structure of the XKR8–BSG complex in lipid nanodiscs

**Figure 1:**
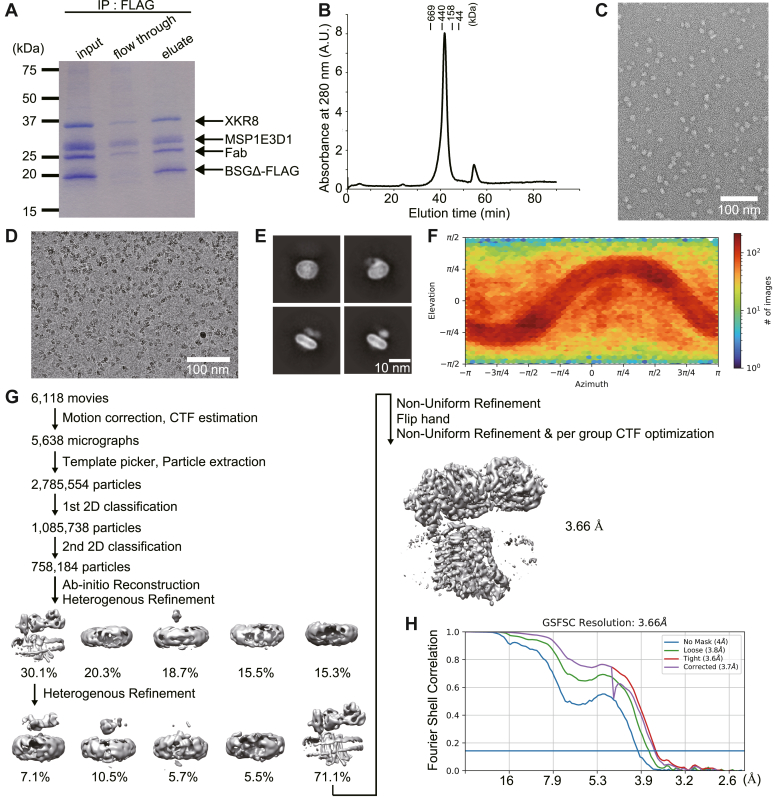
**Single-particle analysis of the human XKR8–basigin complex in lipid nanodiscs.***A*, the purified human XKR8–BSGΔ-FLAG–Fab complex in lipid nanodiscs (29 μg of protein) was analyzed by immunoprecipitation using an anti-FLAG antibody. An aliquot (one-fifth of the input, one-fourth of the flow-through, and two-fifths of the eluate) was separated by SDS-PAGE and stained with CBB. *Arrows* indicate XKR8, MSP1E3D1, Fab, and BSGΔ-FLAG. *B*, the purified human XKR8–BSGΔ-FLAG–Fab complex in lipid nanodiscs (6 μg of protein) was analyzed by gel filtration using Superose 6. The molecular weight of standard proteins is shown in kilodalton. *C*, and *D*, representative negative-staining EM (*C*) and cryo-EM (*D*) images of the purified human XKR8–BSGΔ-FLAG–Fab complex in lipid nanodiscs. *E*, representative 2D class averages generated by the 2D classification. *F*, angular distribution of particles. The number of particles for each orientation is shown as a heat map. Elevation, the vertical angle measured from the horizontal plane; Azimuth, the horizontal angle measured from a reference direction. *G*, data processing workflow. *H*, gold-standard Fourier shell correlation (GSFSC) curve used for the overall resolution estimation. CBB, Coomassie brilliant blue; XKR8, XK-related 8.

The hXKR8–hBSGΔ-FLAG complex was expressed and purified as previously described (17) and reconstituted into lipid nanodiscs, consisting of soybean polar lipids and the membrane scaffold protein MSP1E3D1 (23). The Fab fragment of an anti–human BSG (hBSG) monoclonal antibody (mAb) was bound to the complex, and the sample was purified by gel filtration. To confirm that the hXKR8–hBSGΔ-FLAG–Fab complex successfully formed in lipid nanodiscs, an aliquot of the sample was subjected to coimmunoprecipitation with an anti-FLAG antibody. As shown in Figure 1*A*, hXKR8, Fab, and MSP1E3D1 coprecipitated with hBSGΔ-FLAG, indicating the formation of the complex. Gel filtration and negative-staining EM showed that the purified hXKR8–hBSGΔ-FLAG–Fab complex in lipid nanodiscs was practically monodisperse (Fig. 1, *B* and *C*).

**Figure 2:**
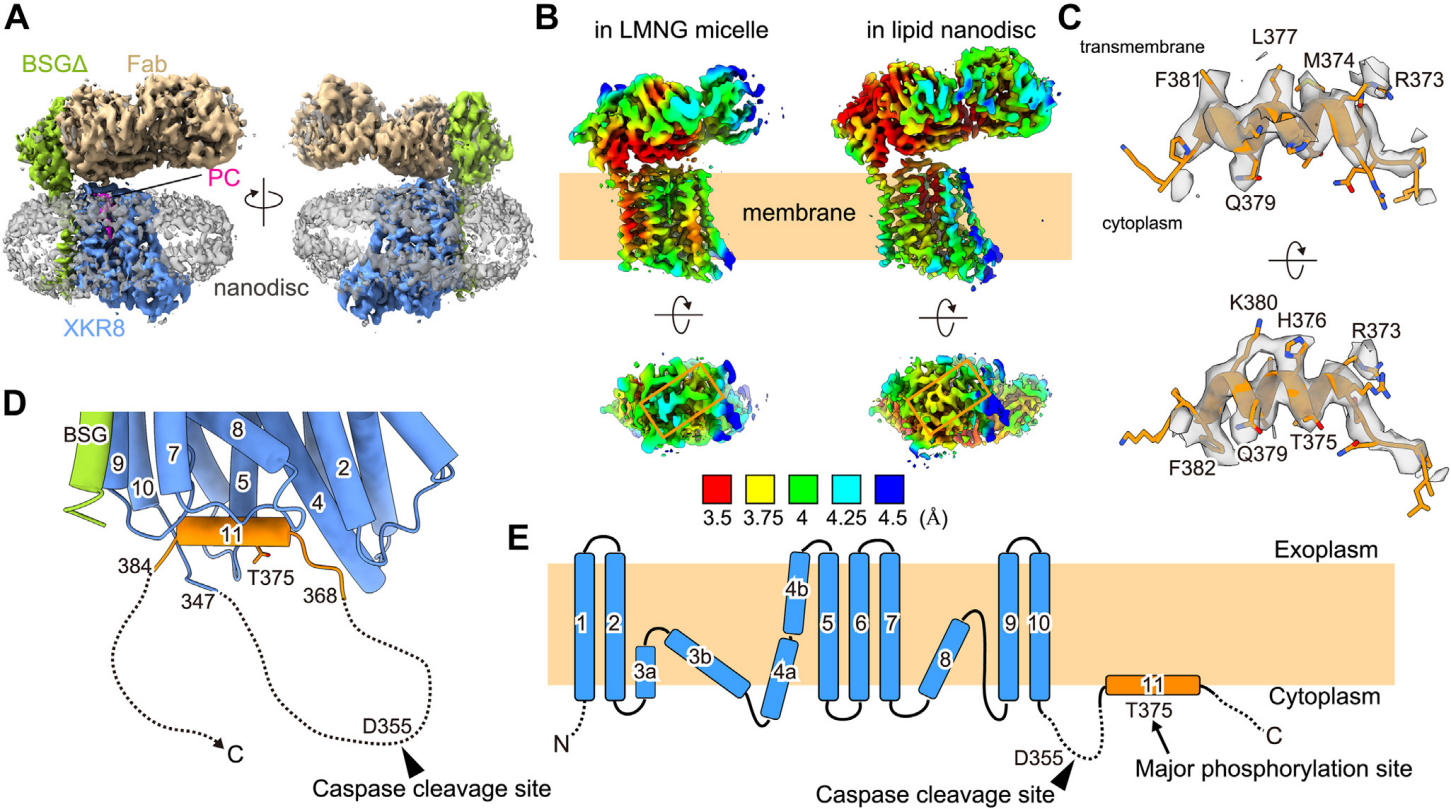
**Revised model of the C-terminal cytoplasmic tail of human XKR8 (hXKR8).***A*, a cryo-EM map of the hXKR8–BSGΔ-FLAG–Fab complex in lipid nanodiscs. *B*, local resolution estimation of the hXKR8–BSGΔ-FLAG–Fab complex map in LMNG micelles (EMD-30636) (*left*) and lipid nanodiscs (*right*). The side (*upper*) and bottom (*lower*) views are shown. The C-terminal cytoplasmic tail region is enclosed by an *orange box*. *C*, side (*upper*) and bottom (*lower*) views of the C-terminal tail region of hXKR8 in lipid nanodiscs, with cryo-EM density (*gray*) superimposed. *D*, a close-up side view of the hXKR8–BSGΔ-FLAG complex around helix 11. Threonine at position 375, the phosphorylation of which activates the scramblase of mXKR8 (16), is shown as a *stick model*. The regions not visualized by the single-particle analysis are depicted as *dotted lines*. An *arrowhead* indicates the caspase cleavage site (D355). *E*, α-Helices of hXKR8 are numbered and schematically shown. The regions not visualized in the present study are shown as *dotted lines*. Caspase cleavage (D355) and putative phosphorylation (T375) sites are indicated by an *arrowhead* and an *arrow*, respectively. BSG, basigin; LMNG, lauryl maltose neopentyl glycol; XKR8, XK-related 8.

The purified sample was vitrified on a holey grid and observed by cryo-EM. Cryo-EM images of the sample showed well-dispersed particles with different orientations (Fig. 1*D*). The single-particle analysis generated a map of the complex at an overall resolution of 3.66 Å (Fig. 1, *E–H*). The complex contained hXKR8, hBSGΔ-FLAG, Fab, and a belt-like structure surrounding the lipid membrane, which appeared to represent MSP1E3D1 (Fig. 2*A*). The overall appearance of the hXKR8–BSG complex in lipid nanodiscs was similar to that observed in lauryl maltose neopentyl glycol (LMNG) micelles (Protein Data Bank [PDB] ID: 7DCE) (Fig. 2*B*). As previously reported in detergent micelles (17), hXKR8 in nanodiscs carried a phospholipid at the cavity on the transmembrane surface (Fig. 2*A*).

### Revised model of the C-terminal tail region of hXKR8

Local resolution estimated with the Phenix program (24) showed that resolution around the cytoplasmic region of hXKR8 was significantly higher in lipid nanodiscs than in LMNG micelles (EMD-30636) (Fig. 2*B*), which may have been due to the stabilization of this region by phospholipids in the nanodiscs. During model building, we noted that the C-terminal cytoplasmic tail of hXKR8 of the previous model (PDB ID: 7DCE) did not fit well with the improved cryo-EM density (Fig. S1*A*). Furthermore, the density map suggested that C-terminal tail after the last transmembrane helix (α10) extended not toward the direction of the C-terminal helix but rather in the cytoplasmic direction, going away from the main body of the protein (Fig. S1*B*). Based on these results, we revised the model of the C-terminal tail as follows. In the previous model, the C-terminal tail after α10 was modeled to extend along the groove in the direction of α2 and α4. In the current model, the C-terminal tail after α10 initially extends in the cytoplasmic direction, is disordered, and then turns along the groove toward the direction of α10. The revised model of this region fits well with cryo-EM density (Fig. 2*C*) and was consistent with the model for rat XKR9 (18). According to the revised model, a disordered loop region following the last transmembrane helix (α10) connected to the C-terminal amphipathic helix (α11), corresponding to amino acids 372 to 382 (Fig. 2, *D* and *E*). The revised model indicated that the caspase-3 cleavage site (amino acid position 355) was located in the disordered loop, which was consistent with all identified caspase cleavage sites mapping to disordered loops (25).

### Interaction between the C-terminal tail and bottom of the transmembrane region

**Figure 3:**
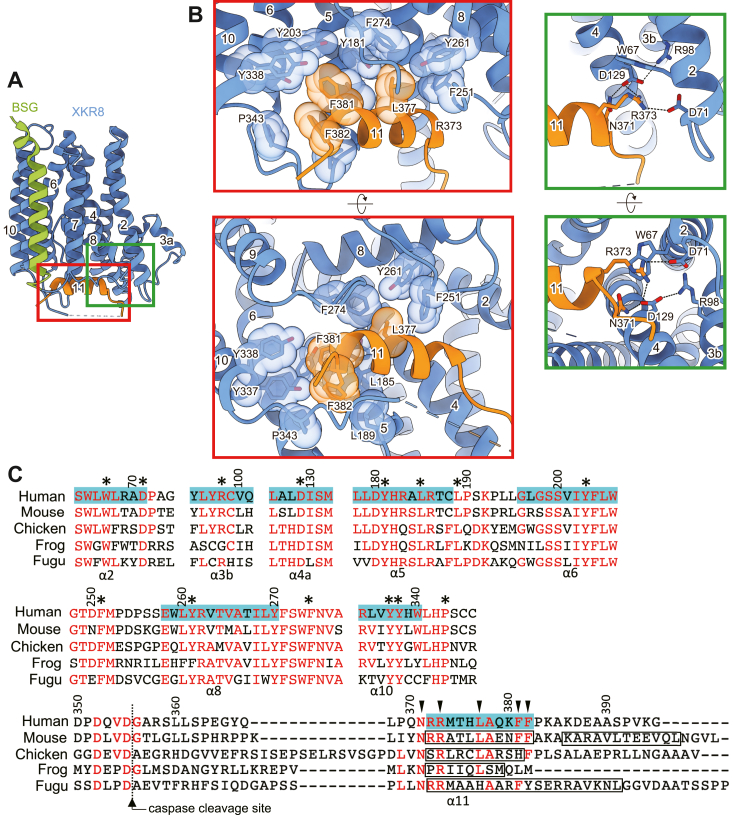
**Interaction of the C-terminal tail of XKR8 and *bottom* of the transmembrane region.***A* and *B*, the structure of the XKR8–basigin (BSG) complex is shown in *A*. The areas enclosed by the *red box* or *green box* in *A* are viewed from the side (*upper*) or bottom (*lower*) in *B*. Residues involved in the interaction are shown as a *stick model* or a *sphere model*. Likely, hydrogen bonds are shown as *dotted lines*. *C*, alignment of the amino acid sequences of XKR8 from human (UniProt: Q9H6D3), mouse (UniProt: Q8C0T0), chicken (UniProt: Q49M60), frog (UniProt: Q49M63), and fugu (UniProt: H2TYQ9). Residues conserved in at least three species are shown in *red*. The *dotted line* marks the caspase cleavage site. α-Helices in hXKR8 are highlighted in *light blue*, and their helix number is shown below the bottom line. The helix in mouse, chicken, frog, and fugu XKR8 corresponding to α11 in human XKR8 is enclosed by *boxes*. Numbers above the first line are the amino acid position of hXKR8. Residues that are present in the *bottom* region of the molecule and interact with α11 in the C-terminal tail region are indicated by *asterisks*. *Arrowheads* indicate the corresponding interacting residues in α11 that were mutated as shown in Figure 4. hXKR8, human XKR8; XKR8, XK-related 8.

The transmembrane region of the hXKR8–BSG complex formed a groove at the cytoplasmic surface (Fig. 3*A*). The C-terminal tail region (amino acid residues from 368 to 384) including α11 of hXKR8 bound to the groove with van der Waals and polar interactions (Fig. 3*B*). The three hydrophobic residues (L377, F381, and F382) on α11 fit into the groove comprising residues in widely different parts of the molecule (L185, L189 [α5], F251 [L7/8], Y261 [α8], F274 [L8/9], Y337, Y338 [α10], and P343 [L10/11]), whereas the two polar residues, N371 and R373, were located closely to the charged residues of D71 and D129, likely forming a tight hydrogen bonding network. In addition, the side chain of W67 appeared to make a van der Waals interaction with the guanidino group of R373. These results suggest that the resting conformation of hXKR8 was tightly maintained by the intense interactions between the C-terminal tail of hXKR8 and the cytoplasmic surface of the transmembrane region. The residues, particularly N371 and R373, are well conserved among XKR8 from several species (human, mouse, chicken, frog, and fugu) (Fig. 3*C*). Similarly, the other amino acid residues involved in this interaction are well conserved (Fig. 3*C*), indicating the important role of these residues in regulating the scrambling activity of XKR8. According to the tertiary model predicted by AlphaFold (26, 27), polar and hydrophobic residues in the C-terminal tail region are likely to interact with residues in the groove at the cytoplasmic surface of the transmembrane in all orthologs (Fig. S2), except for fugu XKR8, in which only polar residues were conserved.

### Regulation of scrambling activity by the C-terminal cytoplasmic tail

**Figure 4:**
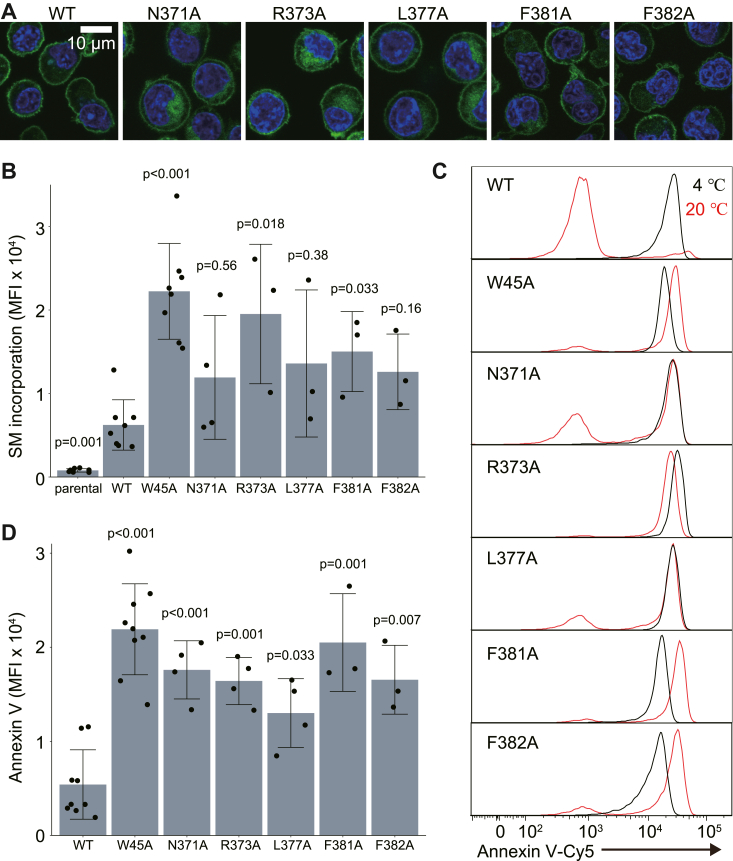
**Regulation of the scrambling activity of XKR8 by the C-terminal helix α11.***A*, Ba/F3 cells expressing the GFP-tagged WT and indicated mutant mXKR8 were observed for GFP (*green*) and Hoechst 33342 (*blue*) by confocal microscopy. *B*, Ba/F3 cells expressing WT or mutant XKR8-GFP were incubated with NBD-SM at 20 °C for 3 min and analyzed by flow cytometry. The mean fluorescence intensity (MFI) of the incorporated NBD-SM is plotted with SD (n ≥3). *C* and *D*, effects of the XKR8 mutation on PtdSer exposure. Ba/F3 cells expressing the WT or mutant mXKR8-GFP were stained with Annexin V-Cy5 at 4 °C or 20 °C for 15 min. *D*, the Annexin V binding experiment at 20 °C was performed at least three times, and MFI was plotted with SD (*bars*). *B* and *D*, the respective scrambling activity of mutant XKR8-GFP was normalized by its expression level (GFP fluorescence) to that of WT XKR8-GFP. Each data point is superimposed on the bar graphs. *p* Values in comparison to the WT were calculated using Student's *t* test with Bonferroni correction. mXKR8, mouse XKR8; PtdSer, phosphatidylserine; XKR8, XK-related 8.

To explore whether the C-terminal tail region hinders scrambling activity in the resting state, the three hydrophobic (L377, F381, and F382) and two polar (N371 and R373) residues in α11 were individually replaced with alanine in mouse XKR8 (mXKR8). The mutants were tagged with GFP at the C terminus and expressed in mouse *TMEM16F*^*−/−*^ *Xkr8*^*−/−*^ Ba/F3 cells in which mXKR8 is activated by phosphorylation (16). As shown in Figure 4*A*, all mutants were present at the plasma membrane, suggesting that the mutations did not markedly affect the protein stability or cellular localization of mXKR8. Parental *TMEM16F*^*−/−*^ *Xkr8*^*−/−*^ Ba/F3 cells did not exhibit scrambling activity assayed by the internalization of fluorescence-labeled SM (NBD-SM) (Fig. 4*B*). When wildtype mXKR8 was expressed, its transformants internalized NBD-SM, confirming that the scramblase activity of mXKR8 was activated in Ba/F3 cells. Consistent with previous findings (17), the scrambling activity of the W45A mutant of mXKR8 was threefold to fourfold stronger than that of the wildtype. All five mutants (N371A, R373A, L377A, F381A, and F382A) in the hydrophobic and polar residues in α11 also considerably enhanced the ability of mXKR8 to internalize NBD-SM by approximately twofold to threefold (Fig. 4*B*), suggesting that these mutations reduced the inhibitory effects of α11 on the scramblase activity of mXKR8.

The impact of the mutation in α11 on scramblase activity was assessed by monitoring the translocation of PtdSer from the inner leaflet to the outer leaflet or the binding of Annexin V to PtdSer exposed on the cell surface. We previously demonstrated that the scramblase activity of mXKR8 is detected at 4 °C, a temperature at which ATP-dependent flippase activity that translocates PtdSer from the outer leaflet to the inner leaflet is reduced (16). Accordingly, *TMEM16F*^*−/−*^ *Xkr8*^*−/−*^ Ba/F3 cell transformants expressing wildtype mXKR8 bound Cy5-labeled Annexin V when the assay was performed at 4 °C; however, this was undetectable at 20 °C (Fig. 4*C*). This absence of binding at 20 °C is attributed to the wildtype mXKR8 activity not surpassing the enhanced flippase activity observed at this temperature.

Conversely, Ba/F3 transformants expressing the W45A mutant bound Annexin V even at 20 °C, which was consistent with its strong scramblase activity detected with the internalization of NBD-SM. Similarly, cells expressing the five mutants (N371A, R373A, L377A, F381A, and F382A) in α11 exposed PtdSer at 20 °C (Fig. 4, *C* and *D*). These results supported the α-helix (α11) in the C-terminal tail suppressing the scramblase activity of XKR8 and the respective mutations releasing this inhibition.

## Discussion

Since XKR8 scramblase is activated when its C-terminal tail is cleaved by a caspase (11) or phosphorylated by a kinase (16), the structure of this region is of particular interest. However, our previous cryo-EM map of the detergent-solubilized XKR8–BSG complex (17) had insufficient local resolution to unambiguously build a model for the C-terminal tail. In the present study, we elucidated the structure of the XKR8–BSG complex embedded in lipid nanodiscs with higher local resolution in the cytoplasmic region, which enabled us to build an unambiguous model of the C-terminal tail region including the amphipathic helix (α11). In the revised model, the caspase recognition site was assigned to the disordered loop between α10 and α11, which was consistent with the notion that caspase recognition sites localize to the disordered loop region (25).

The revised model revealed that the C terminus of hXKR8 bound by both polar and van der Waals interactions with the groove at the cytoplasmic side of the molecule in a similar manner to a “plug” (Fig. 3*B*). The mutational analysis supported the critical role of these interactions in inhibiting the scramblase activity of XKR8 in the resting state (Fig. 4, *B*–*D*). The primary phosphorylation site of XKR8 that affects the scramblase is Thr-375 facing the cytoplasm on the C-terminal α-helix (Fig. 2*D*). The resultant phosphothreonine (Phospho-T375) may form a hydrogen bond with Gln-370 (Fig. S3). Similar to previous findings on the interaction of Myc with Max (28) and model peptides of the α-helix (29, 30), this may destabilize the α-helix, thereby disrupting the interaction between α11 and the bottom region of XKR8 in order to activate its scramblase activity.

The cryo-EM structure of rat XKR9 indicated that the C-terminal tail of XKR9 also interacts with the groove at the cytoplasmic side of the molecule *via* both polar and van der Waals interactions (18) (Fig. S4). The arrangement of polar interactions around the C terminus in XKR9 was similar to that in hXKR8, supporting the crucial roles of these interactions in regulating the scrambling activities of XKR8 and XKR9. In addition to XKR8 and XKR9, XKR4 has been shown to function as a caspase-dependent scramblase (12) and was found to carry a cluster of hydrophilic amino acids followed by hydrophobic residues downstream of the caspase recognition site (Fig. S5). The residues at the bottom region of the molecule interacting with the C-terminal tail region were well conserved among XKR4, XKR8, and XKR9, indicating that these caspase-dependent scramblases apply a similar activation mechanism for their scrambling activity.

We recently demonstrated that XK, a paralog of XKR8/XKR9, functions as a scramblase (31). XK does not have the caspase-recognition sequence at the C terminus (Fig. S5) and is not activated by caspases (12). It was shown to associate with the lipid transporter VPS13A *via* a β-hairpin structure at the bottom of the molecule (31, 32) and is activated by an unknown mechanism in response to the signal from the P2X7 ATP receptor. The residues in the bottom of the molecule that are responsible for the interaction with the C-terminal segment in XKR8 were not well conserved in XK (Fig. S5), suggesting that XK uses a different mechanism from XKR8 or XKR9 to regulate its scramblase activity. On the other hand, it is important to note that XKR7 in the C-terminal tail region carried a caspase-recognition site followed by a hydrophilic–hydrophobic motif similar to those of XKR4 (Fig. S5). The residues likely interacting with these motifs in the bottom region were also conserved between XKR4 and XKR7 (Fig. S5), suggesting that even though PtdSer-scramblase activity is not detected (12), XKR7 may function as a caspase-dependent scramblase for unidentified substrates or uncharacterized conditions.

Our new model revealed that the C-terminal tail associated with the cytoplasmic ends of several transmembrane helices, including α2, α4, and α5, in the resting state (Fig. 3*B*), and also indicated that caspase-mediated cleavage or kinase-mediated phosphorylation induced its dissociation from the bottom of XKR8. As we previously proposed, we expected the dissociation of the C terminus from the bottom of the molecule to rearrange the segments of α1–α5, generating a gap between α1 and α5 for the passage of phospholipids (17). Therefore, it is important to note that local resolution around α1 and α2 in the hXKR8 complex embedded in lipid nanodiscs was lower than that in other regions, implying that these helices are intrinsically dynamic (33) (Fig. 2*B*). Further analyses of the structure that captures the active state or open state of XKR8 and functional analyses are needed to clarify the mechanisms underlying lipid scrambling. The active mutant identified in the present study will provide an essential tool.

## Experimental procedures

### Cell lines, recombinant proteins, and materials

*Spodoptera frugiperda* (Sf) 9 cells (American Type Culture Collection CRL-1711) were cultured in Sf-900Ⅲ SFM medium (Gibco) and *TMEM16F*^*−/−*^*Xkr8*^*−/−*^ Ba/F3 cells (16) in RPMI1640 containing 10% fetal calf serum and 45 units/ml of IL-3.

An hBSG mAb (clone: XBA18) and its Fab fragment were previously described (17). Cy5-labeled Annexin V was from BioVision. LMNG and fluorinated Fos-choline 8 were from Anatrace. Soybean Polar Lipid Extract was from Avanti. Cholesteryl hemisuccinate (CHS) and tobacco etch virus (TEV) protease fused to a histidine tag were from Sigma–Aldrich. *N*-dodecyl-β-d-maltopyranoside was from Dojindo. Methyl-β-cyclodextrin (MβCD) was from Tokyo Chemical Industry Co. A recombinant MSP1E3D1 protein was produced in *Escherichia coli* using its expression plasmid (Addgene) and was purified essentially according to a previously reported protocol (34).

### Expression plasmids

Expression plasmids for hXKR8-TH-GFP and hBSGΔ-FLAG were previously described (17). In hXKR8-TH-GFP, the complementary DNA for hXKR8 was fused at its C terminus with a TEV-cleavage sequence, followed by eight histidine residues (8His), a c-Myc tag, 8His, and monomeric (m) enhanced GFP (mEGFP). hBSGΔ-FLAG lacked the first immunoglobulin-like domain (Ig1) of hBSG and carried the mutated second Ig domain in which asparagine in two N-glycosylation sites (NGS and NGT) at positions 152 and 186 was replaced by glutamine. A FLAG tag was also added to the C terminus.

Mutants of mXKR8 (N371A, R373A, L377A, F381A, and F382A) were prepared by PCR with pPEF-mXkr8-mEGFP-FLAG (16) as the template using the following partially complementary mutagenesis primers and common reverse (5′-acagattctcgaattcgaggactcc-3′) and forward primers (5′-gtgaggaattggatccatcatgc-3′). N371A: 5′-gctgatttatGCcaggcgtgccaccctg-3′ and 5′-ctgGCataaatcagcttaggaggacgatgg-3′ (mutated residues are uppercases and complementary regions are underlined); R373A: 5′-ataacaggGCtgccaccctgttagcagagaac-3′ and 5′-tggcaGCcctgttataaatcagcttaggaggacg-3′; L377A: 5′-cctgGCTgcagagaacttcttcgccaagg-3′ and 5′-ttctctgcAGCcagggtggcacgcctg-3′; F381A: 5′-cagagaacGCcttcgccaaggccaaagctc-3′ and 5′-cgaagGCgttctctgctaacagggtggcac-3′; and F382A: 5′-gagaacttcGCcgccaaggccaaagctcg-3′ and 5′-ggcgGCgaagttctctgctaacagggtggc-3′. The resultant PCR products were introduced into pPEF-mEGFP-FLAG using In-Fusion HD Cloning Kits (Takara Bio), and their authenticity was confirmed by sequencing.

### Purification of the hXKR8–hBSGΔ-FLAG complex

The hXKR8–hBSGΔ-FLAG complex was purified as previously described (17) with minor modifications. In brief, recombinant hXKR8-TH-GFP and hBSGΔ-FLAG were coexpressed in Sf9 cells using a baculovirus-mediated transduction system. Cells were collected 60 h after infection, washed, and suspended in 20 mM Hepes–NaOH buffer (pH 7.0) containing 150 mM NaCl, 1 mM EDTA, 1 mM EGTA, 0.4 mM Pefabloc SC (Merck), 1 mM *p*APMSF, 0.2 mM Tris(2-carboxyethyl)phosphine (TCEP), a protease inhibitor mixture (cOmplete, EDTA-free; Roche Diagnostics), and 1.3 units/ml benzonase (Merck). After the disruption of cells by sonication, samples were centrifuged at 6000*g* for 25 min to remove cell debris. Membrane fractions were then collected by centrifugation at 99,650*g* for 1 h, homogenized by a Dounce homogenizer in 20 mM Hepes–NaOH buffer (pH 7.0) containing 150 mM NaCl, 25% glycerol, 50 mM imidazole, 0.4 mM Pefabloc SC, 1 mM *p*APMSF, 0.2 mM TCEP, and cOmplete EDTA free, and then subjected to sonication. After the addition of LMNG and CHS at final concentrations of 1 and 0.1%, respectively, the solution was rotated at 4 °C for 2 h to solubilize the proteins. Insoluble materials were removed by centrifugation at 99,650*g* for 1 h, and the lysate was loaded onto the HisTrap High-Performance column (Cytiva) connected to the HPLC system (Prominence; Shimadzu). The column was washed with an increasing concentration (60–80 mM) of imidazole in buffer A (20 mM Hepes–NaOH buffer [pH 7.0], 0.01% LMNG, 0.001% CHS, 150 mM NaCl, and 0.2 mM TCEP) containing 25% glycerol. The hXKR8-TH-GFP/hBSGΔ-FLAG complex was eluted using a linear gradient of imidazole concentrations, ranging between 80 and 400 mM, in buffer A with 25% glycerol. It was then subjected to an overnight treatment at 4 °C with TEV protease during dialysis against buffer A containing 10% glycerol and 1 mM EDTA. To remove His-tagged GFP and His-tagged TEV protease, the sample was reloaded to a HisTrap column equilibrated with 20 mM Hepes–NaOH buffer (pH 7.0), 0.005% LMNG, 0.0005% CHS, 150 mM NaCl, 10% glycerol, and 0.2 mM TCEP. Proteins in the flow-through fractions were concentrated to 4 mg/ml by ultrafiltration using an Amicon Ultra-15 50K filter.

### Nanodisc assembly

hXKR8–hBSGΔ-FLAG was embedded into lipid nanodiscs as previously described (35) with some modifications. Soybean polar lipid extract in chloroform was dried under a nitrogen gas stream and kept overnight in a desiccator. Dried lipids were solubilized in buffer B (20 mM Hepes–NaOH [pH 7.0], 100 mM NaCl, 1 mM EGTA, and 0.2 mM TCEP) containing 2% *N*-dodecyl-β-d-maltopyranoside at a final concentration of 10 mg/ml. hXKR8–hBSGΔ-FLAG (2.4 nmol) was incubated by rotating at 4 °C for 30 min in 240 μl of buffer B with 24 nmol MSP1E3D1 and 480 nmol lipids. MβCD was added at a molar ratio of MβCD: detergent of 12:1 and incubated at 30 °C for 10 min to remove the detergents. Insoluble materials were removed by centrifugation at 20,000*g* at 4 °C for 20 min. The efficient incorporation of the hXKR8–hBSGΔ-FLAG complex into the nanodisc suggested that the presence of the FLAG-peptide at the C terminus of hBSGΔ had minimal impact on this integration process.

### Preparation of the hXKR8–hBSGΔ-FLAG–Fab complex in nanodiscs

The hXKR8–hBSGΔ-FLAG complex in nanodiscs was incubated overnight with the Fab fragment of the XBA18 anti-hBSG mAb (17) at 4 °C and subjected to gel filtration using the Superose 6 Increase 10/300 GL column (Cytiva) connected to the HPLC system. The column was developed with buffer B. Fractions containing the hXKR8–hBSGΔ-FLAG–Fab complex in nanodiscs were collected, and proteins were concentrated to 8 mg/ml by ultrafiltration using Vivaspin 2-100K (Cytiva), flash-frozen in liquid nitrogen, and stored at −80 ˚C.

Regarding coimmunoprecipitation, samples were diluted with wash buffer (20 mM Hepes–OH [pH 7.0], 100 mM NaCl, and 1 mM EGTA) and loaded onto anti-FLAG M2 magnetic beads (Sigma–Aldrich) at 4 °C for 2 h. Beads were washed twice with wash buffer, and proteins were eluted with wash buffer containing 150 ng/μl of the 3× FLAG peptide (Sigma–Aldrich). Aliquots from each step were mixed with a 0.25 volume of 5× SDS sample buffer (200 mM Tris–HCl buffer [pH 6.8], 10% SDS, 25% glycerol, 5% β-mercaptoethanol, and 0.05% bromophenol blue), boiled at 95 °C for 5 min, and separated by SDS-PAGE using a 10 to 20% polyacrylamide gel (Nacalai Tesque). Proteins were visualized by Coomassie Brilliant Blue staining.

### Negative-staining EM

Negative-staining EM was performed as previously described (17). In brief, a 3 μl aliquot of the purified hXKR8–hBSGΔ-FLAG–Fab complex in nanodiscs (0.01 mg/ml) was applied to freshly glow-discharged, carbon-coated 200 mesh copper grids (Nisshin EM). After blotting with filter paper (Whatman No. 1), samples were stained with 2% uranyl acetate, blotted completely, and observed by transmission EM (Tecnai G20; FEI) at 200 kV.

### Cryo-EM

In cryo-EM, fluorinated Fos-choline 8 was added at a final concentration of 0.075% to the hXKR8–hBSGΔ-FLAG–Fab complex in lipid nanodiscs. An aliquot (3 μl) of the sample was applied to a glow-discharged Quantifoil holey carbon grid (Au, R1.2/1.3, 300-mesh), blotted for 4 s (+10 blot force, 10 s wait time, and 100% humidity at 6 °C), and plunge-frozen by Vitrobot (Thermo Fisher Scientific). Movies were acquired using a Titan Krios G3i (Thermo Fisher Scientific) equipped with a K3 summit direct electron detector (Gatan) running at 300 kV operated in the counting mode with a nominal magnification of 105,000×, corresponding to a calibrated pixel size of 0.83 Å. Data were automatically acquired using EPU software (Thermo Fisher Scientific). The dose rate was 10.432 e^−^/Å^2^ per second, and the total exposure time was 4.79 s, resulting in a total dose of 50 e^−^/Å^2^ over 50 frames. A total of 6118 movies were collected.

### Cryo-EM data processing

Cryo-EM data were processed using cryoSPARC, version 4.2.1 (Structura Biotechnology Inc) (36). Raw movies were motion-corrected by patch motion correction, and contrast transfer function (CTF) parameters were estimated by patch CTF estimations. Micrographs with a CTF parameter lower than 4Å were removed, resulting in 5638 high-quality micrographs. At first, 1000 micrographs were randomly selected and subjected to Blob Picker with a particle diameter of 100 to 150 Å, and particles were extracted with a box size of 380 pixels and Fourier-cropped to 256 pixels (1.23 Å/pixel). The resultant 235,172 particles were subjected to two rounds of 2D classification, and three 2D class averages with different orientations were selected as templates for template-based picking.

A total of 5638 high-quality micrographs were then subjected to Template Picker, and 2,785,554 particles were extracted. Following this, two rounds of 2D classification were applied to eliminate particles lacking discernible protein features. The remaining 758,184 particles underwent *ab initio* reconstruction with 5 classes, 0.1 similarity, and an initial alignment resolution of 35 Å. Using the resultant five maps as references, the particles in all classes were subjected to heterogenous refinement (20 Å initial low pass). This refinement resulted in the formation of one class exhibiting reasonable features of the XKR8 complex and four classes lacking features of the complex. These additional classes could indicate either empty nanodiscs or nanodiscs containing the XKR8 complex but failing alignment because of partial or complete denaturation of the proteins. The class with reasonable protein features consisting of 228,535 particles was selected and subjected to another round of heterogenous refinement (20 Å initial low pass) using the best-resolved class from the first heterogenous refinement and the four poorly resolved classes from the *ab initio* reconstruction as references. A total of 162,528 particles were selected and subjected to nonuniform refinement (30 Å initial low pass) (37). After flipping the handedness of the volume, another round of nonuniform refinement with the optimization of per-group CTF parameters was performed, yielding a map with an overall resolution of 3.66 Å, estimated by the gold-standard FSC = 0.143 criteria calculated by cryoSPARC. The processing strategy is described in Figure 1*G*. Local resolution estimations were performed in Phenix (24).

### Model building and refinement

The hXKR8–hBSGΔ-FLAG–Fab complex structure in LMNG micelles (PDB ID: 7DCE) was used as a starting model. Regarding the C-terminal tail region of hXKR8, the corresponding region of the AlphaFold model of hXKR8 (Q9H6D3) was fit to the map. The model was then manually fit to the map using Coot (38) and subjected to real-space refinement in Phenix (39). The residues 1 to 6, 348 to 367, and 385 to 395 of hXKR8 were not modeled. All molecular graphics were prepared using UCSF Chimera X (40).

### Establishment of stable transformants and the phospholipid scrambling assay

*TMEM16F*^*−/−*^*Xkr8*^*−/−*^ Ba/F3 cells expressing mouse BSG (16) were transfected with the Ahd1-cleaved pPEF expression plasmid for the mutant mXKR8-tagged C-terminally with mEGFP by electroporation using NEPA21 (Nepa Gene) and cultured in medium containing 0.5 mg/ml G418 and 1.0 μg/ml puromycin. Stable transformants were observed by confocal microscopy (FV-1000D; Olympus) for the cellular localization of mXKR8-GFP.

Phospholipid scrambling activity was measured by PtdSer exposure or the internalization of NBD-SM as previously described (17). In brief, cells were washed with Annexin V buffer (10 mM Hepes–NaOH buffer [pH 7.4], 140 mM NaCl, and 2.5 mM CaCl_2_), incubated on ice or at 20 °C for 15 min with 1000-fold diluted Cy5-Annexin V and 5 μg/ml propidium iodide in Annexin V buffer, and analyzed by flow cytometry (FACSCanto II; BD Biosciences). To assay the incorporation of NBD-SM, cells were washed with Annexin V buffer and incubated at 20 °C for 3 min with 0.5 μM NBD-SM at a cell concentration of 1.0 × 10^6^/ml in Annexin V buffer. A 150 μl aliquot was mixed with an equal volume of Annexin V buffer containing 5 mg/ml fatty acid–free bovine serum albumin (Sigma–Aldrich) and 5 nM SYTOX red (Thermo Fisher Scientific). The amount of the incorporated NBD-SM, which was not extracted with the fatty acid–free bovine serum albumin, was quantified by FACSCanto II. Fluorescence without NBD-SM was subtracted as the background.

## Data availability

The cryo-EM density map for the hXKR8–hBSGΔ-FLAG–Fab complex in nanodiscs was deposited in the Electron Microscopy Data Bank (accession number: EMD-38291). The hXKR8–hBSGΔ-FLAG–Fab complex in nanodisc coordinates was deposited in PDB (accession number: 8XEJ). All other data are contained within the article.

## Supporting information

This article contains supporting information.

## Conflict of interest

The authors declare that they have no conflicts of interest with the contents of this article.
