# Supplementary material for: The role of the C-terminal tail region as a plug to regulate XKR8 lipid scramblase
Source: J Biol Chem. 2024 Feb 15;300(3):105755. doi: 10.1016/j.jbc.2024.105755 (PMC10938166; doi:10.1016/j.jbc.2024.105755)
Supplement: Supplemental Figs. S1–S5 [file mmc1.pdf]

The role of the C-terminal tail region as a plug to regulate XKR8 lipid scramblase

Takaharu Sakuragi[1], Ryuta Kanai[2], Mayumi Otani[1], Masahide Kikkawa[3], Chikashi Toyoshima[2], and Shigekazu Nagata[1*]

[1]Laboratory of Biochemistry and Immunology, World Premier International Immunology Frontier Research Center, Osaka University, Suita, 565-0871 Osaka, Japan

[2]Institute for Quantitative Biosciences, The University of Tokyo, 113-0032 Tokyo, Japan

[3]Department of Cell Biology and Anatomy, Graduate School of Medicine, The University of Tokyo, 113-0033 Tokyo, Japan

Supporting Information:

- **Figure S1.** Interpretation of the map at the cytoplasmic region of XKR8
- **Figure S2.** Interaction of the C-terminal tail of frog XKR8 and the bottom of the transmembrane region.
- **Figure S3.** Provisional model of the C-terminal cytoplasmic tail after the phosphorylation of T375.
- **Figure S4.** Interaction of the C-terminal tail of XKR9 and the bottom of the transmembrane region.
- **Figure S5.** Alignment of amino acid sequences of the human XKR family.

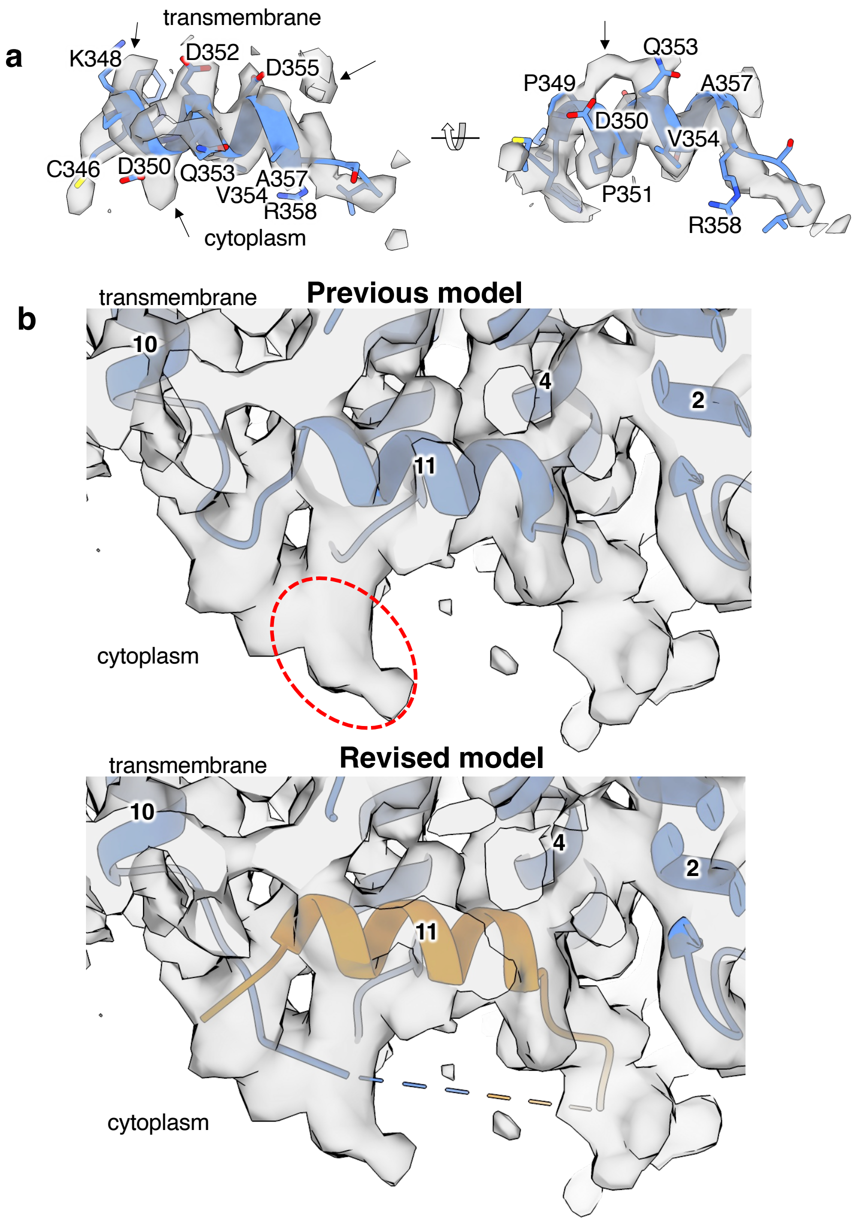

Figure S1. Interpretation of the map at the cytoplasmic region of XKR8. a, Side (left) and bottom (right) views of the C-terminal tail region of the previous XKR8 model (PDB: 7DCE) fit in the current cryo-EM map (grey). Extra densities that are not explained by the previous model are indicated by arrows. **b**, The current Cryo-EM map at a low contour level with the previous (PDB: 7DCE) or revised model (PDB: 8XEJ). Extra density extending towards the cytoplasm that is not explained by the previous model is indicated by a dotted circle.

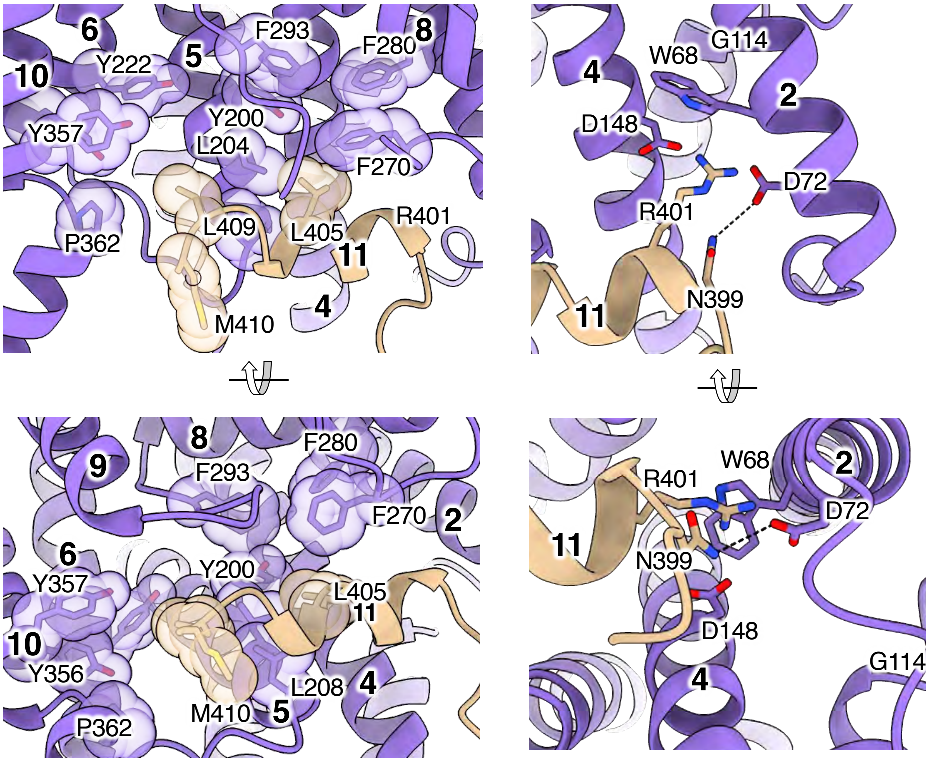

Figure S2. Interaction of the C-terminal tail of frog XKR8 and the bottom of the transmembrane region. The AlphaFold model of frog XKR8 viewed from the side (upper) or bottom (lower). The C-terminal tail region is colored in tan. Residues involved in van der Waals interactions are shown as a stick and sphere model (left). Residues involved in polar interactions are shown as a stick model (right). A likely hydrogen bond is shown as a dotted line.

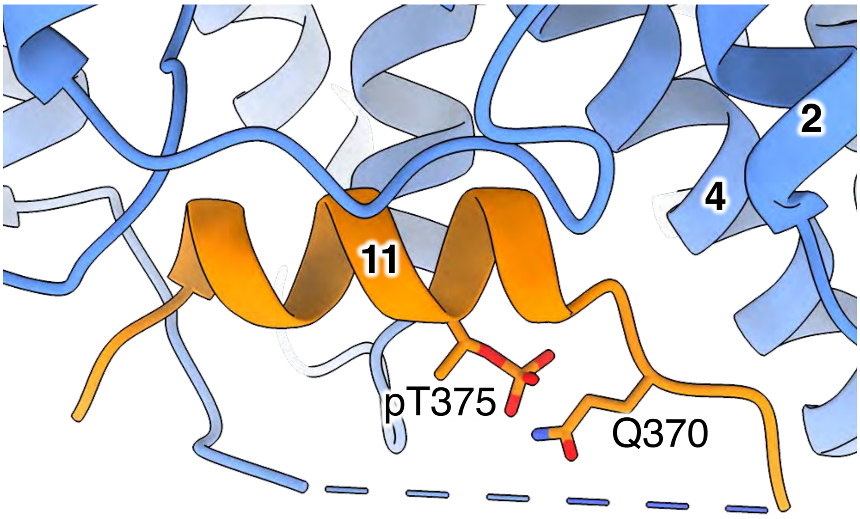

Figure S3. Provisional model of the C-terminal cytoplasmic tail after the phosphorylation of T375. Phosphorylation was introduced into T375 of the model of hXKR8 in lipid nanodiscs (PDB: 8XEJ) by Coot. Phosphorylated T375 (pT375) is assumed to interact with Q370.

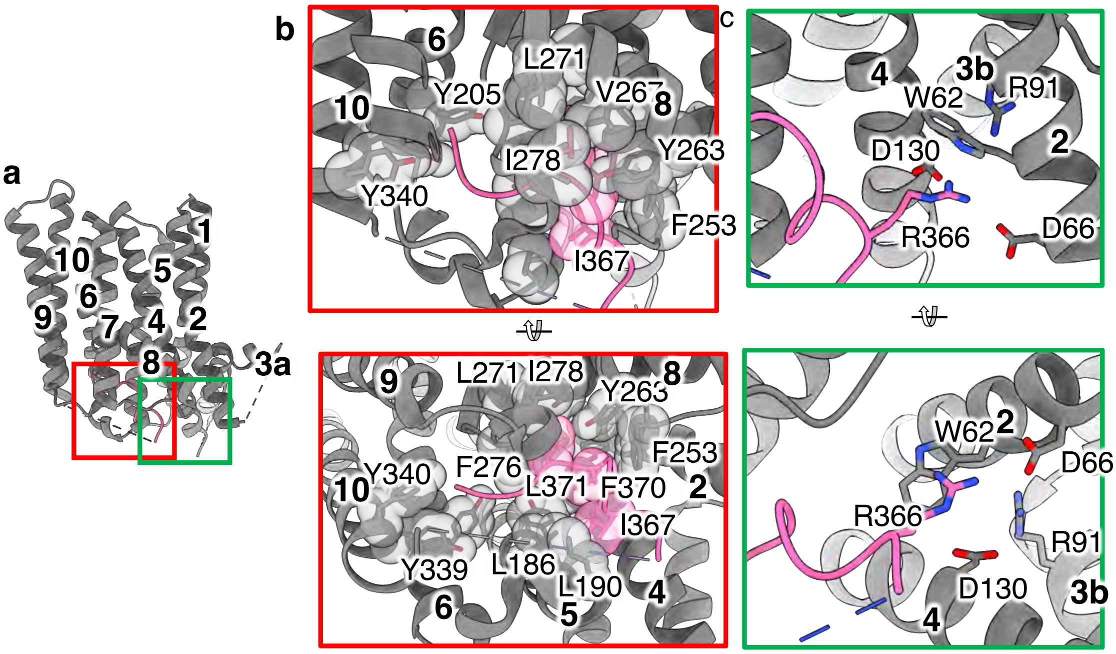

Figure S4. Interaction of the C-terminal tail of XKR9 and the bottom of the transmembrane region. a, The structure of rat XKR9 (PDB 7P14). The C-terminal tail region is colored in pink. **b**, The areas enclosed by the red box in **a** are viewed from the side (upper) or bottom (lower). Residues involved in van der Waals interactions are shown as a stick and sphere model. **c**, The areas enclosed by the green box in **a** are viewed from the side (upper) or bottom (lower). Residues involved in the polar interaction are shown as a stick model.

```
hXkr4    MAAKSDGRLKMKKSSDVAFTPLQNSDHSGSVQGLAPGLPSGSGAEDEEAAGGGCCPDGGGCSRCCCCCAGSGGSAGSGGSGGVAGPGGGGAGSAA
hXKR7    MAAKSDG-------------------------------AAASASPDPEGAAGGARGSAG----------GRGEAAAAAGPPGVVGAGGPGP----

hXK      -------------MKFPASVLASVFLFVAETTAALSLSSTYRSGGDRMWQALTLLFSLLPCALVQL-TLLFVHRDLS------RDR---------
hXkr4    LCLRLGREQRRYSLWDCLWILAAVAVYFADVGTDVWLAVDYYLRGQRWWFGLTLFFVVLGSLSVQVFSFRWFVHDFSTEDSATAAAASSCPQPGA
hXKR7    ----------RYELRDCCWVLCALLVFFSDGATDLWLAASYYLQNQHTYFSLTLLFVLLPSLVVQLLSFRWFVYDYS--------EPAGSPGPAV
hXkr8    ----MPWSSRGALLRDLVLGVLGTAAFLLDLGTDLWAAVQYALGGRYLWAALVLALLGLASVALQLFSWLWLRADPAGLHGSQPPR---------
hXkr9    ---------MKYTKQNFMMSVLGIIIYVTDLIVDIWVSVRFFHEGQYVFSALALSFMLFGTLVAQCFSYSWFKADLKK--AGQESQ---------
                                 α1                                    α2

hXK      ---------------------------------------------------------------PLVLLLHLLQLGPLFRCFEVFCIYFQSGNNEE
hXkr4    DCKTVVGGGSAAGEGEARPSTPQRQASNASKSNIAAANSGSNSSGATRASGKHRSASCSFCIWLLQSLIHILQLGQIWRYFHTIYLGIRSRQSGE
hXKR7    STKDSVAGGAA---------ISTKDSAGAFRTKEGSPEPGPQP--APSSASAYRRRCCRLCIWLLQTLVHLLQLGQVWRYLRALYLGLQSRWRGE
hXkr8    ---------------------------------------------------------------RCLALLHLLQLGYLYRCVQELRQGLLVWQQEE
hXkr9    ---------------------------------------------------------------HCFLLLHCLQGGVFTRYWFALKRGYHAAFKYD
                                                                          α3a            α3b

hXK      PYVSITKKRQMPKNGLSEEIEKEVGQAEGKLITHRSAFSRASVIQAFLGSAPQLTLQLYISV-MQQDVTVGRSLLMTISLLSIVYGALRCNILAI
hXkr4    NDRWRFY---------------------WKMVYEYADVSMLHLLATFLESAPQLVLQLCIIV-QTHSLQALQGFTAAASLVSLAWALASYQK-AL
hXKR7    RLRRHFY---------------------WQMLFESADVSMLRLLETFLRSAPQLVLQLSLLVHRGGAPDLLPALSTSASLVSLAWTLASYQK-VL
hXkr8    PSEFDLA----------------------YADFLALDISMLRLFETFLETAPQLTLVLAIML-QSGRAEYYQWVGICTSFLGISWALLDYHR-AL
hXkr9    SNTSNFV--------------EEQIDLHKEVIDRVTDLSMLRLFETYLEGCPQLILQLYILL-EHGQANFSQYAAIMVSCCAISWSTVDYQV-AL
                                                     α4a              α4b              α5

hXK      KIKYDEYEVKVKPLAYVCIFLWRSFEIATRVVVLVLFTSVLKTWVVVIILINFFSFFLYPWILFWCSGSPFPENIEKALSRVGTTIVLCFLTLLY
hXkr4    RDSRDDKKPI-SYMAVIIQFCWHFFTIAARVITFALFASVFQLYFGIFIVLHWCIMTFW---IVHCETEFCITKWEEIVFDMVVGIIYIFSWFN-
hXKR7    RDSRDDKRPL-SYKGAVAQVLWHLFSIAARGLAFALFASVYKLYFGIFIVAHWCVMTFW---VIQGETDFCMSKWEEIIYNMVVGIIYIFCWFN-
hXkr8    RTCLPSKPLL-GLGSSVIYFLWNLLLLWPRVLAVALFSALFPSYVALHFLGLWLVLLLW---VWLQGTDFMPDPSSEWLYRVTVATILYFSWFN-
hXkr9    RKSLPDKKLLNGLCPKITYLFYKLFTLLSWMLSVVLLLFLNVKIALFLLLFLWLLGIIW---AFKNNTQFCTCISMEFLYRIVVGFILIFTFFN-
                                 α6                        α7                               α8

hXK      TGINMFCWSAVQLKIDSPDLISKSHNWYQLLVYYMIRFIENAILLLLWYLFKTDIYMYVCAPLLVLQLLIGYCTAILFMLVFYQFFHPCKKLFSS
hXkr4    --------------------VKEGRTRCRLFIYYFVILLENTALSALWYLYKAPQIADAFAIPALCVVFSSFLTGVVFMLMYYAFFHPNGPRFG-
hXKR7    --------------------VKEGRSRRRMTLYHCIVLLENAALTGFWYSSRNFS-TDFYSLIMVCVVASSFALGIFFMCVYYCLLHPNGPMLGP
hXkr8    --------------------VAEGRTRGRAIIHFAFLLSDSILLVATWVTHSSWLPSGIPLQLWLPVGCGCFFLGLALRLVYYHWLHPSCCWKPD
hXkr9    --------------------IKGQNTKCPMSCYYIVRVLGTLGILTVFWVCPLTIFNPDYFIPISITIVLTLLLGILFLIVYYGSFHPNRSAETK
                                            α9                                    α10

hXK      SVSEGFQRWLRCFCWACRQQKPCEPIGKEDLQSSRDRDETPSSSKTSPEPGQFLNAEDLCSA---------------------------------
hXkr4    QSPSCACEDPAAAFTLPPDVATSTLRSISNNRSVVSDRDQKFAERDG---CVPVFQVRPTAPSTPSSRPPRIEESVIKIDLFRNRYPAWERHVLD
hXKR7    QAPGCIFRKASEPCGPPADAITSPPRSLP--RTTGAERDGASAGERAGTPTPPVFQVRPGLPPTPVARTLRTEGPVIRIDLPRKKYPAWDAHFID
hXkr8    PDQVDGARSLLSPEGYQLPQNRRMTHLAQKFFPKAKDEAASPVKG--------------------------------------------------
hXkr9    CDEIDGKPVLRECRMRYFLME--------------------------------------------------------------------------
                                 α11

hXkr4    RSLRKAILAFE-CSPSPPRLQYKDDALIQERLEYETTL
hXKR7    RRLRKTILALEYSSPATPRLQYRSVGTSQELLEYETTV
```

Figure S5. Alignment of amino acid sequences of the human XKR family. The amino acid sequences of human XK (P51811), XKR4 (Q5GH76), XKR7 (Q5GH72), XKR8 (Q9H6D3), and XKR9 (Q5GH70) were aligned by introducing several gaps to obtain maximum homology. Amino acids that were identical among at least four members are shown in red. Eleven α-helices are shadowed and numbered, and β-sheets are highlighted in red. Caspase-recognition sites are highlighted in yellow. In the cytoplasmic tail region, a motif of polar residues followed by hydrophobic residues, expected to function as a plug, is highlighted in green. The amino acids in the bottom of the molecules interacting with the C-terminal plug are highlighted in blue.
